# Negative effect of prolonged cecal intubation time on adenoma detection in female patients

**DOI:** 10.1002/jgh3.12861

**Published:** 2023-01-24

**Authors:** Yasuhiko Hamada, Kyosuke Tanaka, Noriyuki Horiki, Junya Tsuboi, Reiko Yamada, Misaki Nakamura, Satoshi Tamaru, Tomomi Yamada, Hayato Nakagawa

**Affiliations:** ^1^ Department of Gastroenterology and Hepatology Mie University Hospital Tsu Japan; ^2^ Clinical Research Support Center Mie University Hospital Tsu Japan; ^3^ Department of Medical Innovation Osaka University Hospital Suita Japan

**Keywords:** adenoma detection, cancer screening, cecal intubation, colonoscopy, colorectal cancer

## Abstract

**Background and Aim:**

Withdrawal time of the colonoscope is associated with adenoma detection. However, the association between cecal intubation time and adenoma detection remains unclear. This study aimed to evaluate the association between cecal intubation time and adenoma detection.

**Methods:**

This retrospective study analyzed prospectively collected data from a randomized controlled trial on female patients who underwent colonoscopy in an academic hospital. The primary outcome was the mean number of all adenomas detected per patient. Secondary outcomes included the mean number of advanced, diminutive, small/large, right‐sided colonic, and left‐sided colonic adenomas detected per patient. Furthermore, the detection rates of all categories of adenoma were evaluated.

**Results:**

The analysis included 216 female patients aged ≥20 years. The correlation analysis did not reveal a significant relationship (*P* = 0.473) between cecal intubation and withdrawal times. The mean number of all adenomas detected per patient declined by approximately 30% (1.05–0.70) from the fastest to the slowest insertion time quartile. Adjusted regression analysis showed a significant decrease in the mean number of all adenomas detected per patient with increased intubation time (relative risk, RR = 0.87; 95% confidence interval, 0.76–0.99, *P* = 0.045), whereas the mean number of other categories of adenomas detected per patient and the detection rates of all categories of adenoma were not associated with the cecal intubation time.

**Conclusions:**

This study showed a significant association between prolonged cecal intubation time and decreased adenoma detection. The cecal intubation time may be a significant quality indicator for colonoscopy.

## Graphical abstract

The association between colonoscope intubation time and adenoma detection is unclear. The current study demonstrated that prolonged cecal intubation time significantly decreased the number of detected adenomas. Cecal intubation time may be an important quality indicator for colonoscopy.

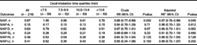

## Introduction

Adenoma detection rate (ADR), defined as the proportion of patients with at least one identified adenoma during colonoscopy, has been considered a significant quality indicator for screening colonoscopy.^1^ Previous studies have shown that a 1% increase in ADR is associated with a 3% and 5% decrease in the incidence and mortality risk of colorectal cancer, respectively.^1^, ^2^ Furthermore, an important quality measure for maximizing ADR is to allow adequate time for mucosal inspection during colonoscope withdrawal. A large community‐based study reported that a withdrawal time of ≥6 min was associated with a higher ADR.^3^ However, prolonging the withdrawal time alone does not guarantee optimal adenoma detection,^4^ suggesting that the technical ability and effort of endoscopists are equally critical.^5^

Traditionally, cecal intubation time has been considered a competency metric for colonoscopy. Previous studies have reported a direct association between a prolonged cecal intubation time and a decreased number of detected adenomas,^6^, ^7^ while others have not found any significant association.^8^, ^9^ Considering these conflicting reports, the association between cecal intubation time and adenoma detection remains unclear. Furthermore, these studies are limited by several factors, including the lack of standards for bowel preparation and the inclusion of therapeutic colonoscopies,^6^, ^7^, ^8^, ^9^ which can potentially cause biases.

Therefore, we aimed to investigate the association between cecal intubation time and adenoma detection using prospectively collected data from a randomized controlled trial (RCT).

## Materials and methods

### Ethics approval and consent to participate

This study was approved by the Ethics Committee of Mie University Hospital on 25 May 2021 (approval number H2021‐095). All procedures were performed in accordance with the guidelines of the responsible institutional and national committee on human experimentation and the Helsinki Declaration of 1964 and its later amendments. Furthermore, the ethics committee of our institution approved the opt‐out method for obtaining consent for the study; consequently, the participants provided informed consent on the institutional website.

### Study population

This study was a post hoc analysis of an RCT investigating the efficacy of a small‐caliber colonoscope in preventing pain during colonoscopy in female patients. The trial was carried out at our institution between October 2013 and November 2017.^10^ The study enrolled 220 female patients ≥20 years of age who underwent non‐therapeutic colonoscopy without sedation. They were randomly assigned in a 1:1 ratio to undergo colonoscopy using a small‐caliber colonoscope (PCF‐PQ260L; Olympus Medical Systems, Tokyo, Japan) or a standard colonoscope (CF‐Q260AI; Olympus Medical Systems). Patients with a history of colorectal surgical resection, pregnancy, inflammatory bowel disease, massive hematochezia, and preference for sedation were excluded from the RCT. Furthermore, the current analysis also excluded patients with incomplete colonoscopy or insufficient documentation of the number of detected adenomas.

### Procedures

Before colonoscopy, the study participants underwent bowel preparation with 2 L of polyethylene glycol solution. Endoscopists assessed the quality of the bowel preparation according to the extent of visible mucosa after suctioning the fluid residue, using the Aronchick Bowel Preparation Scale (excellent, good, fair, poor, or inadequate).^11^ Each patient underwent a colonoscopy only once. The study procedures were performed by 12 endoscopists, each with over 5 years of colonoscopy experience. Patients referred from other hospitals for diagnostic colonoscopy were not included in the study; therefore, the endoscopists had no information on previous colonoscopy or its findings for any of the participating patients. During colonoscopy, a soft distal transparent cap (small‐caliber colonoscope: model MAJ‐1988, outer diameter 10.75 mm; standard colonoscope: model MAJ‐1990, outer diameter 12.75 mm, Olympus Medical Systems) was attached so that 2 mm extended beyond the tip of the colonoscope. Retroflexion to improve adenoma detection in the right‐sided colon was performed at the discretion of the endoscopist. The polyp pathology was examined via biopsy during colonoscopy or endoscopic resection at a later date.

### Outcome measurement

The primary outcome was the mean number of all adenomas detected per patient (MAP), which is a more valid representation of adenoma detection than ADR.^12^ Secondary outcomes included the mean number of advanced adenomas (MAP‐A), diminutive adenomas (MAP‐D; ≤5 mm in diameter), small/large adenomas (MAP‐SL; >5 mm in diameter), right‐sided colonic adenomas (MAP‐Rt), and left‐sided colonic adenomas (MAP‐Lt) detected per patient. Furthermore, ADR, advanced ADR (A‐ADR), diminutive ADR (D‐ADR), small/large ADR (SL‐ADR), right‐sided colonic ADR (Rt‐ADR), and left‐sided colonic ADR (Lt‐ADR) were also evaluated. The right‐side colon included the cecum, ascending colon, and transverse colon, whereas the left‐side colon included the descending colon, sigmoid colon, and rectum. ADR was defined as the proportion of patients in whom at least one adenoma was detected. An advanced adenoma was defined as a tubular adenoma with a diameter ≥10 mm, villous component, or high‐grade dysplasia.

Cecal intubation time was defined as the time from the insertion of the tip into the rectum to the time until passing a point proximal to the ileocecal valve so that the base of the cecum was visible. Withdrawal time was defined as the time taken to withdraw the tip of the colonoscope from the base of the cecum to the anus, which includes the time spent observing and sampling the detected polyps for biopsy.

### Statistical analysis

The following analyses were conducted to examine the association between cecal intubation time and adenoma detection. The cecal intubation times of all patients were categorized into cecal intubation time quartiles. The number of detected adenomas and the ADR in each cecal intubation time quartile were assessed. Continuous variables were expressed as mean and standard deviations, and compared using Student's *t*‐test or analysis of variance (ANOVA). Categorical variables were expressed as numbers and proportions, and compared using Pearson's chi‐squared test or Fisher's exact test, as appropriate. The relationship between cecal intubation and withdrawal times was evaluated by correlation analysis. The Poisson regression analysis was applied to examine the association between cecal intubation time and MAP, MAP‐A, MAP‐D, MAP‐SL, MAP‐Rt, and MAP‐Lt. The results are expressed as relative risk (RR) with a 95% confidence interval (CI). Logistic regression analysis was used to examine the association between cecal intubation time and ADR, A‐ADR, D‐ADR, SL‐ADR, Rt‐ADR, and Lt‐ADR. The results are expressed as odds ratio (OR) with a 95% CI. Subsequently, a multivariate regression analysis was performed to examine the adjusted RR or OR of the cecal intubation time quartiles with 95% CI. All regression models considered age, previous abdominal surgery (absence or presence), bowel preparation quality (excellent, good, fair, poor, or inadequate), type of colonoscope (standard or small‐caliber), and withdrawal time quartiles (withdrawal time of <8.6 min, 8.6–10.9 min, 11.0–14.2 min, or >14.2 min) as possible cofounders. All statistical analyses were performed using IBM SPSS Statistics version 26 (IBM Corp., Armonk, NY, USA). All tests were two‐sided, and statistical significance was established at *P* < 0.05.

## Results

**Figure 1:**
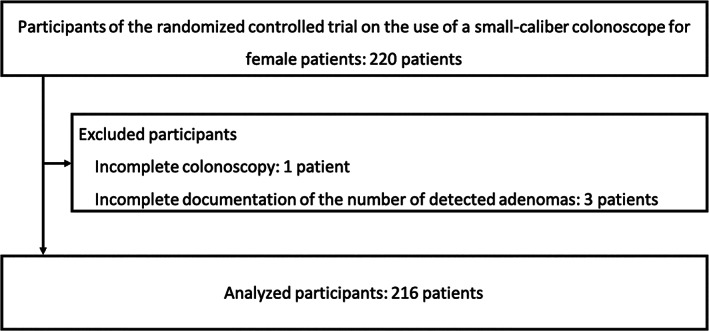
Patient selection process.

The patient selection process is presented in Figure 1. Of the 220 patients originally enrolled in the RCT, 4 patients, including 1 with an incomplete colonoscopy and 3 with incomplete documentation of the number of detected adenomas, were excluded. Finally, data from 216 patients were analyzed in this study.

### Baseline characteristics according to the cecal intubation time

**Figure 2:**
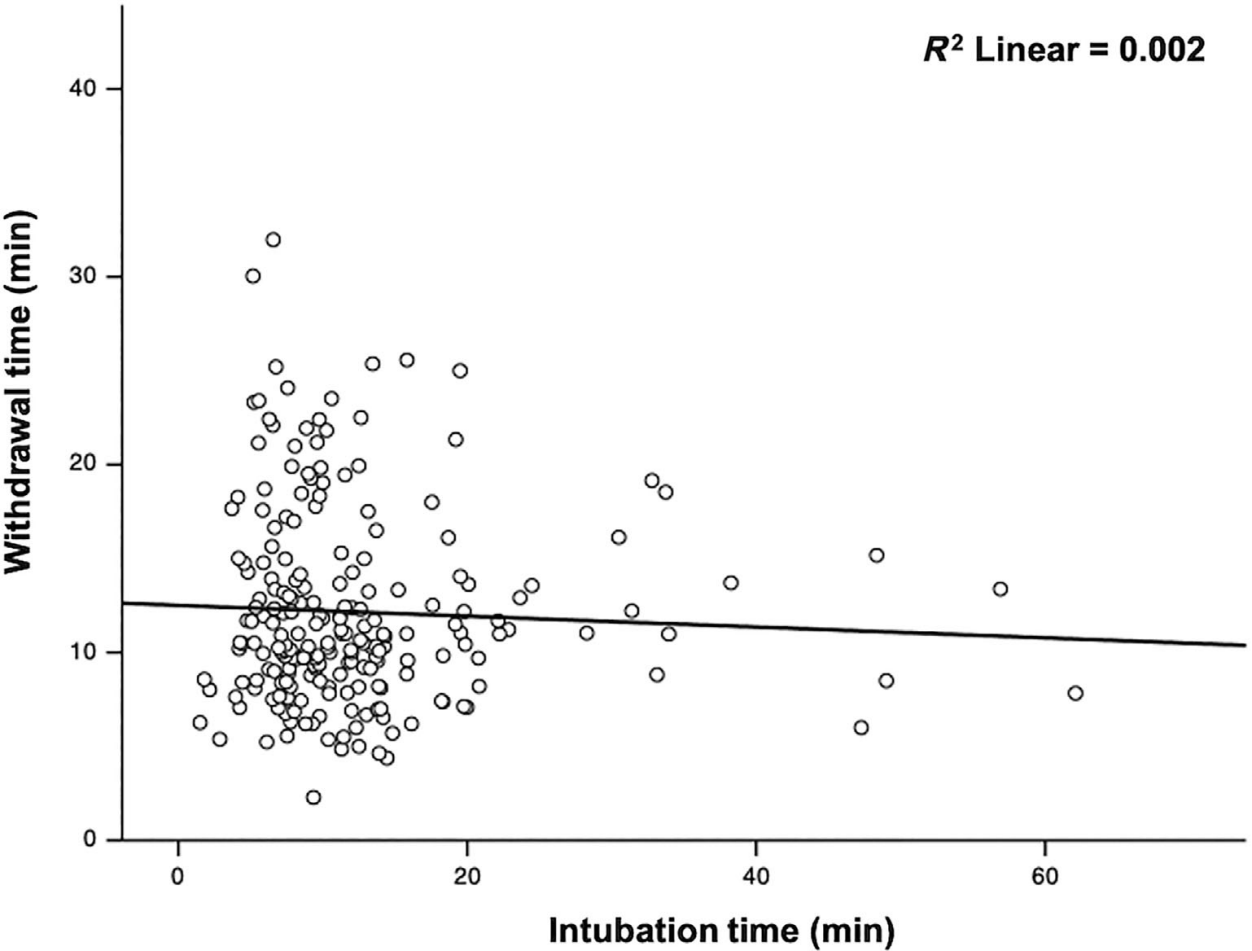
Relationship between cecal intubation time and colonoscope withdrawal time. *P* = 0.473 on correlation analysis.

Patient and procedural characteristics according to the cecal intubation time quartiles are shown in Table 1. Age, indication for colonoscopy, bowel preparation quality, previous abdominal surgery, type of colonoscope, and withdrawal time were not significantly different across quartiles. Furthermore, the correlation analysis revealed a lack of relationship (*P* = 0.473) between cecal intubation and withdrawal times (Fig. 2).

**Table 1:** Clinical characteristics according to cecal intubation time quartiles

	All (*n* = 216)	Cecal intubation time quartiles (min)				*P*‐value
		<7.5 (*n* = 58)	7.5–9.9 (*n* = 52)	10.0–13.8 (*n* = 53)	>13.8 (*n* = 53)	
Age, mean (SD), years	62.9 (12.9)	59.9 (13.5)	64.2 (12.1)	64.2 (12.8)	63.7 (13.4)	0.226
Indication for colonoscopy, *n* (%)						0.774
Screening	44 (20.4)	10 (17.2)	8 (15.4)	12 (22.6)	14 (26.4)	
Surveillance	39 (18.0)	12 (20.7)	8 (15.4)	10 (18.9)	9 (17.0)	
Diagnosis	135 (61.6)	32 (62.1)	36 (69.2)	31 (58.5)	30 (56.6)	
Bowel preparation quality, *n* (%)						0.653
Excellent	65 (30.1)	18 (31.0)	19 (36.5)	19 (36.5)	16 (30.2)	
Good	110 (50.9)	32 (55.2)	22 (42.3)	22 (42.3)	26 (49.0)	
Fair	41 (19.0)	8 (13.8)	11 (21.1)	11 (21.2)	11 (20.8)	
Poor	0 (0.0)	0 (0.0)	0 (0.0)	0 (0.0)	0 (0.0)	
Inadequate	0 (0.0)	0 (0.0)	0 (0.0)	0 (0.0)	0 (0.0)	
Previous abdominal surgery, *n* (%)						0.377
No	98 (45.4)	31 (53.4)	25 (48.1)	21 (39.6)	21 (39.6)	
Yes	118 (54.6)	27 (46.4)	27 (51.9)	32 (60.4)	32 (60.4)	
Type of colonoscope, *n* (%)						0.252
Standard	107 (49.5)	24 (41.4)	25 (48.1)	26 (49.1)	32 (60.4)	
Small‐caliber	109 (55.5)	34 (58.6)	27 (51.9)	27 (50.9)	21 (39.6)	
Cecal intubation time, mean (SD), min	12.3 (8.8)	5.7 (1.4)	8.7 (0.8)	12.0 (1.1)	23.4 (11.5)	<0.001
Withdrawal time, mean (SD), min	12.2 (5.2)	13.0 (5.9)	12.3 (5.2)	11.8 (4.8)	11.4 (4.6)	0.379

### Association between cecal intubation time and the number of detected adenomas

The association between cecal intubation time and MAP is shown in Table 2. MAP decreased from 1.05 to 0.70 from the fastest to the slowest insertion time quartile. Furthermore, adjusted regression analysis showed that there was a significant decrease in MAP with increased cecal intubation time (RR, 0.87; 95% CI, 0.76–1.00, *P* = 0.045). Although not statistically significant, MAP‐D tended to decrease with increased cecal intubation time (RR, 0.86; 95% CI, 0.73–1.01, *P* = 0.062). No significant associations were noted between the cecal intubation time and MAP‐A, MAP‐SL, MAP‐Rt, and MAP‐Lt.

**Table 2:** Association between cecal intubation time and the mean number of detected adenomas per patient

Outcomes	All (*n* = 216)	Cecal intubation time quartiles (min)				Crude RR (95% CI)	*P‐*value	Adjusted RR^†^ (95% CI)	*P*‐value
		<7.5 (*n* = 58)	7.5–9.9 (*n* = 52)	10.0–13.8 (*n* = 53)	>13.8 (*n* = 53)				
MAP, *n*	0.87	1.05	0.88	0.81	0.70	0.88 (0.77–0.99)	0.042	0.87 (0.76–0.99)	0.045
MAP‐A, *n*	0.15	0.17	0.15	0.13	0.15	0.94 (0.70–1.28)	0.71	0.96 (0.70–1.32)	0.812
MAP‐D, *n*	0.63	0.79	0.62	0.60	0.51	0.87 (0.75–1.01)	0.07	0.86 (0.73–1.01)	0.062
MAP‐SL, *n*	0.24	0.26	0.29	0.21	0.19	0.89 (0.69–1.13)	0.33	0.91 (0.70–1.19)	0.490
MAP‐Rt, *n*	0.46	0.53	0.54	0.38	0.38	0.64 (0.73–1.04)	0.125	0.86 (0.72–1.04)	0.126
MAP‐Lt, *n*	0.41	0.52	0.35	0.43	0.32	0.55 (0.34–1.06)	0.183	0.88 (0.72–1.07)	0.202

^†^ Relative risk was adjusted for age, previous abdominal surgery, type of colonoscope, bowel preparation quality, and withdrawal time quartiles.

CI, confidence interval; MAP, mean number of all adenomas detected per patient; MAP‐A, mean number of advanced adenomas detected per patient; MAP‐D, mean number of diminutive adenomas (≤5 mm in diameter) detected per patient; MAP‐Lt, mean number of left‐sided colonic adenomas detected per patient; MAP‐Rt, mean number of right‐sided colonic adenomas detected per patient; MAP‐S, mean number of small/large adenomas (>5 mm in diameter) detected per patient; RR, relative risk.

### Association between cecal intubation time and ADR

The association between the cecal intubation time quartiles and ADR is shown in Table 3. Adjusted regression analyses showed that none of the ADRs significantly decreased with an increase in cecal intubation time.

**Table 3:** Association between cecal intubation time quartiles and adenoma detection rates

Outcomes	All (*n* = 216)	Cecal intubation time quartiles (min)				Crude OR (95% CI)	*P‐*value	Adjusted OR^†^ (95% CI)	*P*‐value
		<7.5 (*n* = 58)	7.5–9.9 (*n* = 52)	10.0–13.8 (*n* = 53)	>13.8 (*n* = 53)				
ADR (%)	42.6	44.8	42.3	41.5	41.5	0.96 (0.75–1.21)	0.713	0.94 (0.72–1.22)	0.624
A‐ADR (%)	11.6	13.8	9.6	11.3	11.3	0.94 (0.65–1.36)	0.751	0.98 (0.87–3.22)	0.936
D‐ADR (%)	37.0	41.4	36.5	37.7	32.1	0.89 (0.70–1.14)	0.356	0.86 (0.66–1.13)	0.276
SL‐ADR (%)	15.7	19.0	17.3	11.3	15.1	0.87 (0.63–1.21)	0.419	0.89 (0.62–1.28)	0.538
Rt‐ADR (%)	29.6	31.0	34.0	26.4	26.4	0.90 (0.70–1.17)	0.435	0.87 (0.65–1.16)	0.349
Lt‐ADR (%)	29.2	34.5	23.1	30.2	28.3	0.94 (0.73–1.22)	0.647	0.93 (0.70–1.23)	0.600

^†^ Odds ratio was adjusted for age, previous abdominal surgery, type of colonoscope, bowel preparation quality, and withdrawal time quartiles.

A‐ADR, advanced adenoma detection rate; ADR, adenoma detection rate; I, confidence interval; D‐ADR, diminutive adenoma (≤5 mm in diameter) detection rate; Lt‐ADR, left‐sided colonic adenoma detection rate; OR, odds ratio; Rt‐ADR, right‐sided colonic adenoma detection rate; SL‐ADR, small/large adenoma (>5 mm in diameter) detection rate.

## Discussion

This study showed a significant association between prolonged cecal intubation time and decreased adenoma detection. MAP declined by approximately 30% (1.05–0.70) from the fastest to the slowest insertion time quartile. Furthermore, the cecal insertion time did not lead to a shorter or longer withdrawal time. These results suggest that the cecal intubation time may be a surrogate marker for adenoma detection, regardless of the length of the withdrawal time, and endoscopists should focus on adequate mucosal inspection in cases of difficult cecal intubation to compensate for fewer detected adenomas.

Considering that cecal insertion time may reflect technical skill, there are a few studies clarifying a possible association between cecal insertion time and adenoma detection.^6^, ^7^ These previous studies support our observation that prolonged cecal intubation impairs the quality of mucosal inspection on withdrawal, resulting in missed polyps. Nevertheless, findings of the previous studies differed from those of our study in some ways. Yang *et al*. reported an inverse association between cecal intubation time and the number of diminutive adenomas detected in patients undergoing screening colonoscopy.^6^ However, the participants were limited to asymptomatic patients, and we consider that our results better reflect real clinical practice than the previous study. Von Renteln *et al*. reported an inverse association between cecal intubation time and the number of adenomas of all sizes detected in patients undergoing elective outpatient colonoscopy.^7^ However, the median withdrawal time of 8.6 min in that study may have been insufficient to corroborate the inverse association, as Kim *et al*. reported that a withdrawal time of <10 min was insufficient to identify all adenomas.^13^ In contrast, our study supports the existence of an inverse association even when sufficient withdrawal time (median time of 12.2 min) is taken.

Patient‐ and endoscopist‐dependent factors are the key determinants of cecal intubation time. Patient‐dependent factors include sex, age, previous abdominal surgery, and bowel preparation quality.^14^, ^15^ Longer intubation time is generally caused by bent and redundant colons, which may obstruct mucosal inspection and consequently decrease the number of detected adenomas. For instance, reaching the cecum through a colonic loop may obstruct the examination of the proximal colon, and difficult intubation through the sigmoid colon may obstruct mucosal inspection during withdrawal of the colonoscope.^16^ The cecal intubation time may also reflect the endoscopist's level of fatigue and concentration. Prolonged cecal intubation can exhaust the endoscopist, causing impaired concentration. Furthermore, it may disrupt the schedule of the endoscopist, resulting in a possible hastened withdrawal with a higher chance of overlooking polyps.^17^, ^18^, ^19^, ^20^

On the contrary, some previous studies have shown no direct relationship between cecal intubation time and the number of detected adenomas.^8^, ^9^ Instead, they have suggested that the cecal intubation time to withdrawal time ratio (IWTR) was a quality indicator of adenoma detection, as IWTR <1.0 was associated with the detection of more adenomas compared with IWTR ≥1.0. These studies proposed using an IWTR cut‐off of 1.0 as a surrogate indicator of proper colonoscopy techniques and skills. However, these studies failed to assess the withdrawal time correctly after excluding the time spent in biopsy sampling or endoscopic resection of the polyps. As the result, their withdrawal times might have been prolonged with an increased number of detected polyps, resulting in a decreased IWTR. Thus, the relationship between the IWTR and adenoma detection must be interpreted cautiously.

Our study was a post hoc analysis of an RCT that investigated the efficacy of a small‐caliber colonoscope (PCF‐PQ260L) in preventing pain during colonoscopy. Therefore, the effect of using the small‐caliber colonoscope on adenoma detection should be considered. The small‐caliber colonoscope can be pressed backward by colonic spasms, which may make endoscopic observation more difficult and result in missed polyps. However, similar to the work reported by Luo *et al*., our original study showed that there was no significant difference between the small‐caliber and standard colonoscope groups in adenoma detection.^10^, ^21^ Thus, we believe that the use of the small‐caliber colonoscope (PCF‐PQ260L) does not impair adenoma detection.

To improve adenoma detection, multiple methods, devices, and artificial intelligence (AI)‐based diagnostic tools have been developed.^22^ Various reports indicate that polyps are more likely to be missed in the right‐sided colon than the left‐sided colon during colonoscopy.^23^, ^24^, ^25^ Retroflexion in the right‐sided colon allows observation from the oral side of the folds, which is known to improve adenoma detection when combined with the forward view.^26^, ^27^ In addition, repeated observation of the right‐sided colon also improves adenoma detection.^28^

Distal attachment devices can reduce the incidence of blind spots, such as the oral side of the folds, during colonoscopy. The Endocuff (Olympus Corporation) has a fixed portion and two rows of eight soft projections to improve adenoma detection.^29^ The projections fold backward during insertion so as not to interfere with insertion, and are pulled forward to hold back and straighten the colonic folds during withdrawal. Endocuff Vision is an improved version of the Endocuff with a row of eight longer and softer projections.^30^, ^31^ A meta‐analysis indicated that adenoma detection was significantly higher during Endocuff Vision‐assisted colonoscopy than during standard colonoscopy.^32^

Advances in AI‐assisted colonoscopy have also been associated with improved adenoma detection. Several studies have reported the performance of AI‐based diagnostic systems using test videos, demonstrating real‐time polyp detection with over 90% sensitivity.^33^, ^34^ Wang *et al*. reported that adenoma detection was significantly higher in the AI‐assisted colonoscopy group than in the control group.^35^ Thus, it can be inferred that AI‐assisted colonoscopy can avoid decreased adenoma detection due to fatigue and decreased concentration in the endoscopist as a result of prolonged cecal intubation.

Our study had several strengths. First, the data from the RCT were collected prospectively, which minimized measurement biases. Second, the use of multiple regression analyses largely eliminated the influence of possible confounders affecting cecal intubation time and adenoma detection. However, our study also had some limitations. First, although RCT data can distinctively define the measurement standards for the factors evaluated, the current study was conducted retrospectively at a single academic hospital. Second, the study was limited to female patients; hence, the sample size was smaller than in previous studies. Therefore, further studies that additionally enroll male patients are required to complement our findings.

## Conclusion

In conclusion, we found that a prolonged cecal intubation time significantly decreased MAP, especially in the detection of diminutive adenomas. Our results indicate that cecal intubation time may be an important quality indicator for colonoscopy. Moreover, when encountering a difficult colonoscopy procedure leading to prolonged cecal intubation time, endoscopists may need to perform a meticulous inspection during withdrawal of the colonoscope to compensate for fewer detected adenomas. Regardless, further studies are required to validate the relationship between cecal intubation time and adenoma detection.

## Data Availability

The datasets generated and/or analyzed during the current study are available from the corresponding author upon reasonable request.

## References

[1] Kaminski MF , Regula J , Kraszewska E *et al*. Quality indicators for colonoscopy and the risk of interval cancer. N. Engl. J. Med. 2010; 362: 1795–803. 20463339 10.1056/NEJMoa0907667

[2] Corley DA , Jensen CD , Marks AR *et al*. Adenoma detection rate and risk of colorectal cancer and death. N. Engl. J. Med. 2014; 370: 1298–306. 24693890 10.1056/NEJMoa1309086 PMC4036494

[3] Barclay RL , Vicari JJ , Doughty AS , Johanson JF , Greenlaw RL . Colonoscopic withdrawal times and adenoma detection during screening colonoscopy. N. Engl. J. Med. 2006; 355: 2533–41. 17167136 10.1056/NEJMoa055498

[4] Corley DA , Jensen CD , Marks AR . Can we improve adenoma detection rates? A systematic review of intervention studies. Gastrointest. Endosc. 2011; 74: 656–65. 21741643 10.1016/j.gie.2011.04.017

[5] Lee RH , Tang RS , Muthusamy VR *et al*. Quality of colonoscopy withdrawal technique and variability in adenoma detection rates (with videos). Gastrointest. Endosc. 2011; 74: 128–34. 21531410 10.1016/j.gie.2011.03.003

[6] Yang MH , Cho J , Rampal S *et al*. The association between cecal insertion time and colorectal neoplasm detection. BMC Gastroenterol. 2013; 13: 124. 23915303 10.1186/1471-230X-13-124 PMC3750659

[7] von Renteln D , Robertson DJ , Bensen S , Pohl H . Prolonged cecal insertion time is associated with decreased adenoma detection. Gastrointest. Endosc. 2017; 85: 574–80. 27590962 10.1016/j.gie.2016.08.021

[8] Fritz CDL , Smith ZL , Elsner J , Hollander T , Early D , Kushnir V . Prolonged cecal insertion time is not associated with decreased adenoma detection when a longer withdrawal time is achieved. Dig. Dis. Sci. 2018; 63: 3120–5. 29721773 10.1007/s10620-018-5100-x

[9] Benson ME , Reichelderfer M , Said A , Gaumnitz EA , Pfau PR . Variation in colonoscopic technique and adenoma detection rates at an academic gastroenterology unit. Dig. Dis. Sci. 2010; 55: 166–71. 19156519 10.1007/s10620-008-0703-2

[10] Hamada Y , Tanaka K , Katsurahara M *et al*. Efficacy of a small‐caliber colonoscope for pain in female patients during unsedated colonoscopy: a randomized controlled study. Endosc. Int Open. 2021; 9: E1055–61. 34222630 10.1055/a-1464-0780 PMC8211489

[11] Kastenberg D , Bertiger G , Brogadir S . Bowel preparation quality scales for colonoscopy. World J. Gastroenterol. 2018; 24: 2833–43. 30018478 10.3748/wjg.v24.i26.2833 PMC6048432

[12] Denis B , Sauleau EA , Gendre I *et al*. The mean number of adenomas per procedure should become the gold standard to measure the neoplasia yield of colonoscopy: a population‐based cohort study. Dig. Liver Dis. 2014; 46: 176–81. 24054769 10.1016/j.dld.2013.08.129

[13] Kim JH , Kim YS , Cheon JH *et al*. Influence of the insertion time and number of polyps on miss rate in colonoscopy. Scand. J. Gastroenterol. 2011; 46: 634–9. 21370993 10.3109/00365521.2011.558111

[14] Jaruvongvanich V , Sempokuya T , Laoveeravat P , Ungprasert P . Risk factors associated with longer cecal intubation time: a systematic review and meta‐analysis. Int. J. Colorectal Dis. 2018; 33: 359–65. 29520457 10.1007/s00384-018-3014-x

[15] Kim HY . Cecal intubation time in screening colonoscopy. Medicine. 2021; 100: e25927. 34106660 10.1097/MD.0000000000025927 PMC8133265

[16] Church J . Adenoma detection rate and the quality of colonoscopy: the sword has two edges. Dis. Colon Rectum. 2008; 51: 520–3. 18322755 10.1007/s10350-008-9239-y

[17] Gurudu SR , Ratuapli SK , Leighton JA , Heigh RI , Crowell MD . Adenoma detection rate is not influenced by the timing of colonoscopy when performed in half‐day blocks. Am. J. Gastroenterol. 2011; 106: 1466–71. 21502998 10.1038/ajg.2011.125

[18] Lee A , Iskander JM , Gupta N *et al*. Queue position in the endoscopic schedule impacts effectiveness of colonoscopy. Am. J. Gastroenterol. 2011; 106: 1457–65. 21448145 10.1038/ajg.2011.87 PMC4098876

[19] Teng TY , Khor SN , Kailasam M , Cheah WK , Lau CC . Morning colonoscopies are associated with improved adenoma detection rates. Surg. Endosc. 2016; 30: 1796–803. 26198158 10.1007/s00464-015-4448-7

[20] Dong Z , Wang J , Chen Y *et al*. Negative effects of endoscopists' fatigue on colonoscopy quality on 34,022 screening colonoscopies. J. Gastrointestin. Liver Dis. 2021; 30: 358–65. 34551036 10.15403/jgld-3687

[21] Luo DJ , Hui AJ , Yan KK *et al*. A randomized comparison of ultrathin and standard colonoscope in cecal intubation rate and patient tolerance. Gastrointest. Endosc. 2012; 75: 484–90. 21963069 10.1016/j.gie.2011.07.032

[22] Ikematsu H , Murano T , Shinmura K . Detection of colorectal lesions during colonoscopy. DEN Open. 2022; 2: e68. 35310752 10.1002/deo2.68 PMC8828173

[23] Brenner H , Hoffmeister M , Arndt V , Stegmaier C , Altenhofen L , Haug U . Protection from right‐ and left‐sided colorectal neoplasms after colonoscopy: population‐based study. J. Natl. Cancer Inst. 2010; 102: 89–95. 20042716 10.1093/jnci/djp436

[24] Singh H , Nugent Z , Demers AA , Kliewer EV , Mahmud SM , Bernstein CN . The reduction in colorectal cancer mortality after colonoscopy varies by site of the cancer. Gastroenterology. 2010; 139: 1128–37. 20600026 10.1053/j.gastro.2010.06.052

[25] Baxter NN , Warren JL , Barrett MJ , Stukel TA , Doria‐Rose VP . Association between colonoscopy and colorectal cancer mortality in a US cohort according to site of cancer and colonoscopist specialty. J. Clin. Oncol. 2012; 30: 2664–9. 22689809 10.1200/JCO.2011.40.4772 PMC3413278

[26] Lee HS , Jeon SW , Park HY , Yeo SJ . Improved detection of right colon adenomas with additional retroflexion following two forward‐view examinations: a prospective study. Endoscopy. 2017; 49: 334–41. 27931050 10.1055/s-0042-119401

[27] Miyamoto H , Naoe H , Oda Y *et al*. Impact of retroflexion in the right colon after repeated forward‐view examinations. JGH Open. 2018; 2: 282–7. 30619938 10.1002/jgh3.12084 PMC6308076

[28] Desai M , Bilal M , Hamade N *et al*. Increasing adenoma detection rates in the right side of the colon comparing retroflexion with a second forward view: a systematic review. Gastrointest. Endosc. 2019; 89: 453–9.e3. 30222971 10.1016/j.gie.2018.09.006

[29] Tsiamoulos ZP , Saunders BP . A new accessory, endoscopic cuff, improves colonoscopic access for complex polyp resection and scar assessment in the sigmoid colon (with video). Gastrointest. Endosc. 2012; 76: 1242–5. 23164515 10.1016/j.gie.2012.08.019

[30] van Doorn SC , van der Vlugt M , Depla A *et al*. Adenoma detection with endocuff colonoscopy versus conventional colonoscopy: a multicentre randomised controlled trial. Gut. 2017; 66: 438–45. 26674360 10.1136/gutjnl-2015-310097

[31] Ngu WS , Bevan R , Tsiamoulos ZP *et al*. Improved adenoma detection with endocuff vision: the ADENOMA randomised controlled trial. Gut. 2019; 68: 280–8. 29363535 10.1136/gutjnl-2017-314889 PMC6352411

[32] Patel HK , Chandrasekar VT , Srinivasan S *et al*. Second‐generation distal attachment cuff improves adenoma detection rate: meta‐analysis of randomized controlled trials. Gastrointest. Endosc. 2021; 93: 544–53.e7. 33031786 10.1016/j.gie.2020.09.045

[33] Misawa M , Kudo SE , Mori Y *et al*. Artificial intelligence‐assisted polyp detection for colonoscopy: initial experience. Gastroenterology. 2018; 154: 2027–9.e3. 29653147 10.1053/j.gastro.2018.04.003

[34] Urban G , Tripathi P , Alkayali T *et al*. Deep learning localizes and identifies polyps in real time with 96% accuracy in screening colonoscopy. Gastroenterology. 2018; 155: 1069–78.e8. 29928897 10.1053/j.gastro.2018.06.037 PMC6174102

[35] Wang P , Berzin TM , Glissen Brown JR *et al*. Real‐time automatic detection system increases colonoscopic polyp and adenoma detection rates: a prospective randomised controlled study. Gut. 2019; 68: 1813–9. 30814121 10.1136/gutjnl-2018-317500 PMC6839720

